# Incorporating health literacy in education for socially disadvantaged adults: an Australian feasibility study

**DOI:** 10.1186/s12939-016-0373-1

**Published:** 2016-06-04

**Authors:** Danielle M. Muscat, Sian Smith, Haryana M. Dhillon, Suzanne Morony, Esther L. Davis, Karen Luxford, Heather L. Shepherd, Andrew Hayen, John Comings, Don Nutbeam, Kirsten McCaffery

**Affiliations:** The Screening and Test Evaluation Program (STEP), Sydney School of Public Health, The University of Sydney, Sydney, NSW 2006 Australia; Centre for Medical Psychology and Evidence-based Decision-making (CeMPED), Sydney School of Public Health, The University of Sydney, Sydney, NSW 2006 Australia; Psychosocial Research Group, Prince of Wales Clinical School, Faculty of Medicine, University of New South Wales, Sydney, NSW 2052 Australia; Centre for Medical Psychology and Evidence-based Decision-making (CeMPED), School of Psychology, The University of Sydney, Sydney, NSW 2006 Australia; Concord Clinical School, The University of Sydney, Sydney, NSW 2006 Australia; Illawarra Institute for Mental Health, School of Psychology, University of Wollongong, Wollongong, NSW 2522 Australia; Patient Based Care, Clinical Excellence Commission, Sydney, NSW 2000 Australia; Psycho-oncology Co-operative Research Group (PoCoG), School of Psychology, The University of Sydney, Sydney, NSW Australia; School of Public Health and Community Medicine, University of New South Wales, Sydney, NSW 2052 Australia; Center for International Education, University of Massachusetts, Amherst, MA 01003 USA; Sydney School of Public Health, The University of Sydney, Sydney, NSW 2006 Australia

**Keywords:** Health literacy, Adult education, Health education, Qualitative, Health inequalities

## Abstract

**Background:**

Adult education institutions have been identified as potential settings to improve health literacy and address the health inequalities that stem from limited health literacy. However, few health literacy interventions have been tested in this setting.

**Methods:**

Feasibility study for an RCT of the UK Skilled for Health Program adapted for implementation in Australian adult education settings. Implementation at two sites with mixed methods evaluation to examine feasibility, test for change in participants’ health literacy and pilot test health literacy measures.

**Results:**

Twenty-two socially disadvantaged adults with low literacy participated in the program and received 80–90 hours of health literacy instruction. The program received institutional support from Australia’s largest provider of vocational education and training and was feasible to implement (100 % participation; >90 % completion; high teacher satisfaction). Quantitative results showed improvements in participants’ health literacy skills and confidence, with no change on a generic measure of health literacy. Qualitative analysis identified positive student and teacher engagement with course content and self-reported improvements in health knowledge, attitudes, and communication with healthcare professionals.

**Conclusions:**

Positive feasibility results support a larger RCT of the health literacy program. However, there is a need to identify better, multi-dimensional measures of health literacy in order to be able to quantify change in a larger trial. This feasibility study represents the first step in providing the high quality evidence needed to understand the way in which health literacy can be improved and health inequalities reduced through Australian adult education programs.

**Electronic supplementary material:**

The online version of this article (doi:10.1186/s12939-016-0373-1) contains supplementary material, which is available to authorized users.

## Background

Health literacy is commonly defined as the capacity to acquire, understand and use information in ways which promote and maintain good health [1, 2]. Up to 60 % of Australian adults lack basic health literacy skills to understand health-related materials, such as instructions on a medicine label [3]. Similarly, prevalence estimates from Europe and the United States suggest 47 % and 36 % of the general population respectively have inadequate or limited health literacy [4, 5] with prevalence increasing among those with lower levels of education, the elderly, adults from ethnic minority groups and those with chronic disease [6]. Low health literacy is independently associated with a wide range of poor health outcomes (e.g. increased mortality, reduced overall health status, poorer adherence to medication and health advice and poorer self-care [7, 8]). Low health literacy is also associated with greater inpatient admissions and emergency department visits and higher emergency department and inpatient department spending [9].

Enhancing health literacy has emerged as a national and global public health priority to reduce health inequity [10–12]. Increasingly, adult education organisations are being recognised as able to generate the changes needed to achieve this goal [13, 14]. Adult education institutions offer programs for adult learners who often have no post-secondary education. They are either community owned and managed or government-funded. In Australia and other countries, adult education institutions often have a national network. They provide services to populations of socially-disadvantaged individuals who are likely to lack health literacy skills [4], but express a desire to learn about health [15, 16]. Incorporating functional health literacy skills development (including, for example, reading and interpreting food and medicine labels) into adult education is a natural extension of the literacy skills that are already taught [17]. Given appropriate resources and infrastructure, adult education organisations can provide adult learners with formal exposure to health knowledge and practices, and build the skills required to obtain and use health information [18].

Approaches to incorporating health literacy into adult education vary widely [14]. Where health content is embedded into literacy programs it tends to be on an ad hoc basis [19]. However, more formal programs beyond single-site instruction exist. The United States *Health Literacy Study Circles* series, for example, is designed to enable teachers to integrate health literacy skills development into existing adult education programs by providing tools for the development of units, lessons and evaluation plans [20]. The United Kingdom *Skilled for Health* program embeds health topics within an adult education framework in an aim to improve both (a) adult health literacy, and (b) foundation skills in literacy, language and numeracy (http://rwp.excellencegateway.org.uk). The evaluation of the *Skilled for Health* program identified advantages of the program in recruiting and retaining socially-disadvantaged individuals into an adult literacy program and showed improvements in self-rated health literacy skills [21].

Few health literacy initiatives have been rigorously evaluated using a randomised design to investigate whether heath literacy within adult education can improve both health literacy and general literacy. Evaluation of the 2006 *Expecting the Best* health literacy program for English as a Second Language (ESL) speakers showed benefits of health literacy training on a purpose-designed curriculum-based measure, with no difference between health literacy and standard ESL groups on a validated measure of health literacy (S-TOFHLA). However, attrition from the program was high, with only 30 % of students completing post-test assessments [22]. In a larger randomised controlled trial of a different health literacy curriculum for Spanish speaking adults in the US, results showed a significantly higher increase in the TOFHLA posttest score in the intervention group [23]. Other trials have provided limited details of health literacy assessment tools and quantitative health literacy gains [24].

In the current study, we adapted the *Skilled for Health* program to meet Australian national health guidelines to be suitable for an adult education setting in Australia, with the aim to evaluate it in a randomised controlled trial. To assess the feasibility of delivering the Australian program in an adult education setting and optimise the final, large-scale trial, we delivered the program (*Being Healthy, Staying Healthy)* in two Australian adult education colleges in 2012. The key aims of this study were to: (a) assess the feasibility of delivering a large-scale health literacy program within Australian adult education for socially disadvantaged adults (see Table 1), (b) examine the potential impact a program of this kind could have on health knowledge and skills, health literacy, and perceived confidence in performing health tasks, (c) pilot measures of health literacy and health skills in this setting in preparation for the main trial. This paper reports on the quantitative and qualitative findings of the study in order to evaluate these key feasibility aims.

**Table 1:** Criteria for success of feasibility

− Ability to implement the program within an established adult education course structure.
− High participation and retention rates.
− Ease of course delivery.
− Content deemed to be appropriate for Australian Stage 2 learners.
− Content deemed (by teachers and students) to be engaging and relevant.
− Adult education teachers feeling well supported and confident to deliver a course focused on health.

## Methods

### Development of the *Being Healthy, Staying Healthy* program

The *Being Healthy, Staying Healthy* program embedded key Learning, Literacy and Numeracy skill development into 29 health-related topics using Functional Context Education methods (an approach to adult learning that embeds functional basic skills within topics that are of relevance and interest to adult learners [25]). The 29 topics were derived from the UK *Skilled for Health* program and reflected Australian health priorities, with 6 mandatory core topics assessed as part of the functional health literacy evaluation. Topics were categorised into either the (a) *Being Healthy* category concerning the management of health conditions; or (b) *Staying Healthy* category pertaining to healthy living for illness and disease prevention. See Table 2. Each of the 29 topics embedded basic-level reading (e.g. reading information from a national immunization program schedule), writing (e.g. labelling the human body), speaking (e.g. participating in Dr-patient consultation role plays), listening (e.g. listening to simulated emergency phone calls) and numeracy (e.g. comparing units of measurement) skill activities using varied teaching methods.

**Table 2:** Course Content and Structure

Day	*Being Healthy* Teacher Manual 1	Day	*Staying Healthy* Teacher Manual 2
1	1.1 Introduction 1.2 Baseline assessment	2	2.1 Getting involved^a^ 2.2 Food groups
3	3.1 Taking temperature^a^ 3.2 Checking medicine labels^a^	4	4.1 Food labels^a^
5	5.1 Prescriptions 5.2 Dosage and timing	6	6.1 Nutritional information^a^
7	7.1 Health workers 7.2 Telling your doctor what is wrong	8	8.1 Food temperature safety 8.2 Food date safety
9	9.1 Asking questions^a^ 9.2 Immunisation and health screening	10	10.1 What is a portion? 10.2 Budgeting
11	11.1 Talking to your doctor 11.2 Answering your doctor’s questions	12	12.1 Understanding a diet 12.2 Drinking enough fluids
13	13.1 Completing medical forms 13.2 Emergency services	14	14.1 Heart rate and pulse
15	15.1 Advice from pharmacist 15.2 Follow written instructions	16	16.1 Being active 16.2 First aid demonstrations
17	17.1 Saving lives	18	18.1 Revision
19	19.1 Following emergency instructions	20	20.1 Post assessment

^a^Core topic

### Participant recruitment and program delivery

The program was delivered at two Technical and Further Education (TAFE) colleges in metropolitan New South Wales (NSW) within basic/beginner level Learning, Literacy and Numeracy courses (Australian Core Skills Framework stage 2 [26]). TAFE NSW is Australia’s largest provider of vocational education and training, funded by the NSW state government. Ethics approval was obtained from the University of Sydney Human Research Ethics Committee and 2 institutes of TAFE NSW. One teacher at each of the 2 participating TAFE sites consented to deliver the program and assessments during semester 1, 2012. All students enrolled in the participating courses were invited by their teacher to join the study and were provided with a participant information statement and consent form.

The program was delivered using an established adult literacy course structure at each centre. All students completed 80–90 h of health literacy classes (centre 1; 8 h per week for 10 weeks: centre 2; 5 h per week for 18 weeks). Once the core topics had been completed, students and teachers could select topics to suit their interest and needs.

### Teaching materials

Teachers were each provided with 2 course manuals corresponding with the *Being Healthy*, and *Staying Healthy* course components to facilitate program delivery. The manuals contained guided lesson plans for each topic, including suggested discussion questions, resources such as diagrams and audio recordings, and suggestions for individual and class activities. Answers were included for teachers’ reference.

### Quantitative data collection methods and measures

Outcomes were evaluated among teachers and students using a mixed-methods pre-post design to; (a) provide an indication of the potential impact an Australian adult education health literacy course could have on health knowledge, skills and health literacy, and (b) confirm the appropriateness of measurement instruments for a larger health literacy trial in this context.

### Demographics

Demographic information (gender, age, level of education, language spoken at home, country of origin, and employment status) was taken from student enrolment forms at the beginning of the semester.

### Functional health literacy skills (course-content specific measure)

Twenty-one specific functional health literacy skills-based questions were developed by the investigators from the core topics of the course. Items assessed students’ ability to interpret core elements of the information from a thermometer, medicine label, food and nutrition label, and an audio-recorded healthcare encounter with a pharmacist. Missing responses were scored 0 and total scores could range from 0 to 25. See Additional file 1 for the full marking scheme.

### Functional, critical and communicative health literacy (generic measure)

The functional, critical and communicative health literacy scales for diabetes [27] were adapted to measure generic health literacy by removing all diabetes-specific terminology. The measure consisted of 5 functional health literacy items, 5 communicative health literacy items, and 4 critical health literacy items. Response options ranged from “Never” (1) to “Always” (4).

### Perceived confidence performing health tasks

Eight items were developed to assess student confidence to perform a range of health-related tasks, such as filling out medical forms and using a thermometer. The items were purpose-designed to match the content of the health literacy course, but were adapted from an existing health literacy measure (health literacy screening questions [28]). Response options ranged from “Not at all” (1) to “Very” (4), with higher scores indicating greater confidence.

### Satisfaction with the course

Four questions assessed student satisfaction with the course (post-intervention only). Students were asked to rate (a) the course overall, (b) the clarity of the course, and (c) how much the course helped them to understand their health. Responses were on a five point Likert scale from “Strongly disagree” (1) to “Strongly agree” (5). Students were also asked to indicate whether they would recommend this course to others on a five point scale from “Yes, definitely” (1) to “Definitely not” (5).

### Qualitative data collection methods and analysis

Semi-structured interviews were conducted with students and teachers 4 to 6 weeks after the course to better understand the experience and challenges of teaching and learning health literacy, attitudes towards course structure and content, and impact of the course on health knowledge and skills.

Interviews were conducted in English at adult learning sites by a researcher trained in qualitative methods (ED), audio-recorded and transcribed verbatim.

Qualitative data was analysed using the 5 key steps of the Framework Analysis method [29]: 1) Familiarisation with the data: DM and SM read a sample of transcripts. 2) Creating a thematic framework: DM, SM, SS and KM collaboratively developed a provisional thematic coding framework to chart the data, based on the research question and recurrent issues identified from participants’ accounts. 3) Indexing: DM coded a selection of the transcripts and met regularly with other researchers to refine the framework. 4) Charting: DM synthesized the data within the final thematic chart (a matrix with participants as rows and themes as columns). 5) Mapping and interpretation: Researchers met regularly to discuss and interpret the interview findings.

## Results

### Quantitative results: program impact and measurement appropriateness

#### Recruitment and retention

All of the 22 students who were invited to participate in the study consented to do so. Of those, 20 students (91 %) participated in the follow-up assessment.

### Sample characteristics

Of the 22 students who participated in the study, the majority were women (*n* = 17) and 20 were from non-English speaking backgrounds (NESB). The majority (*n* = 18) were not employed and 7 reported having no formal educational qualification from high school or above. Participants were aged 49 years on average (*SD* = 10.1; *Range* = 29–68). See Table 3.

**Table 3:** Student Demographic Characteristics

Characteristic	*n*	%
Gender (*N* = 22)		
Female	17	77.3
Male	5	22.7
Education (*N* = 20)		
Diploma, bachelor or higher degree	3	15.0
TAFE certificate	8	40.0
School or intermediate certificate	2	10.0
No formal qualification	7	35.0
Language spoken at home (*N* = 21)		
English	1	4.8
Non-English	20	95.2
Country of origin (*N* = 21)		
Australia	1	4.8
Asia	12	57.1
Africa and Middle East	3	14.3
Other	5	23.8
Employment status (*N* = 21)		
Part-time	3	14.3
Unemployed – seeking full-time work	4	19.0
Unemployed – seeking part-time work	1	4.8
Unemployed – not seeking employment	13	61.9

### Functional health literacy skills (course-content specific measure)

Overall the results of the functional health literacy assessment suggest that students’ skills improved. Total mean scores on the functional health literacy skills assessment increased from 12.8 (*SD* = 5.1; 95 % CI [10.5, 15.0]) at baseline to 19.3 (*SD* = 3.6; 95 % CI [17.5, 21.0]) upon completion of the course. The proportion of correct answers increased for 20 of the 22 items. (See Table 4).

**Table 4:** Functional Health Literacy Skills: Number and percentage correct at baseline and follow-up

	Baseline *n*(%) correct *N* = 22	Follow-up *n*(%) correct *N* = 20	Difference %
Temperature			
What temperature is shown?	13 (59)	18 (90)	31
Is the temperature shown ok for a healthy adult?	17 (77)	19 (95)	18
What temperature is normal for a healthy adult?	14 (64)	17 (85)	21
Medicine labels			
When does this medicine expire?	14 (64)	15 (75)	11
I have a stomach ulcer. Can I use this medicine?	16 (73)	14 (70)	-3
My son is 6 years old. Can he use this medicine?	18 (82)	17 (85)	3
I take 2 capsules at 10 am. When can I take 2 more?	14 (64)	16 (80)	16
What should you do if a 3 year old eats 6 capsules?	15 (68)	18 (90)	22
Food and nutrition labels			
What is this packaged food?	14 (64)	19 (95)	31
What is the main ingredient of this soup?	3 (14)	12 603)	46
How do you make this soup?	17 (77)	18 (90)	14
Would you buy this package next July?	12 (55)	14 (70)	15
What are kilojoules (KJs)?	1 (05)	9 (45)	40
How many KJs are in a serve of this soup?	6 (27)	15 (75)	48
How do you find out if this food is high in salt?	9 (41)	11 (55)	14
Why would this be important to check?	1 (05)	4 (20)	15
How could you make sure this food was healthy?	8 (36)	11 (55)	19
Oral communication			
Number of tablets	14 (64)	19 (95)	31
Dosage and timing I	17 (77)	16 (80)	3
Dosage and timing II	18 (82)	16 (80)	-2
What to do if you did not understand the pharmacist	9 (41)	18 (90)	49
Total mean functional health skills score (SD)	12.8 (5.1)	19.3 (3.6)	

### Functional, critical and communicative health literacy (generic measure)

There was very little change in mean total functional, critical and communicative health literacy scores from baseline (*M* = 36.7, *SD* = 5.9, 95 % CI [33.9, 39.5]) to follow-up (*M* = 35.9, *SD* = 4.2, 95 % CI [33.8, 38.1]).

### Perceived confidence performing health tasks

The proportion of students indicating that they were “Mostly” or “Very” confident performing individual functional health tasks increased from baseline to follow-up on 7 of the 8 items, with the percentage increase ranging from 4 % to 59 %. See Table 5

**Table 5:** Confidence performing individual functional health tasks

	Baseline selecting mostly/very		Follow-up selecting mostly/very		Difference
	*N*	*n* (%)	*N*	*n* (%)	%
Confidence					
Telling the doctor what is wrong	20	13 (65)	19	11 (58)	-7
Understanding the doctor	20	6 (30)	18	16 (89)	+59
Reading and understanding medicine labels	20	5 (25)	18	12 (67)	+42
Filling out medical forms	18	4 (22)	18	6 (33)	+11
Preventing problems with your health	17	9 (53)	19	15 (79)	+26
Reading and understanding food labels	19	10 (53)	18	15 (83)	+30
Planning a healthy eating plan	19	11 (58)	18	14 (78)	+20
Using a thermometer	18	12 (67)	17	12 (71)	+4

### Satisfaction with the course

Fourteen of the 20 students who completed the follow-up assessment rated the program as “Excellent” or “Very good” and that the course was clear and easy to understand. Almost all students reported they would recommend the course to others (*n* = 19) and that the course helped them to better understand their health (*n* = 18).

### Qualitative results: feasibility and acceptability of delivering a health literacy program

Qualitative interviews with students and teachers suggest that the course was appropriate for basic/beginner adult learners and feasible to implement in an adult education setting. Specific themes identified across classes included: (a) perceived impact of the course (b) student engagement and perceptions; (c) challenges in participation and delivery and (d) teaching experience and recommendations.

Students’ (S) verbatim quotations are followed by participant number, gender (M or F), age and English language status (NESB or English speaker [ES]). Teachers’ (T) verbatim quotations are followed by their participant number.

### Perceived impact

#### Knowledge and functional health literacy skills

Students had limited or no previous formal experience learning about issues regarding their health. They felt positively about the impact of the course on health knowledge (e.g. knowledge of body systems, and the content of medicine and nutrition labels) and skills (e.g. reading medicine and nutrition labels and taking temperatures)

*“… before [the course] I can’t understand for anything for health, what I do, what I can eat… Before I no [sic] understand medicine, but now…I can, I can.” (S118, F, 61, NESB)*

Knowledge, skills and key concepts acquired during the course were used to help and advise friends, family and members of the extended community. Information passed on to others primarily concerned how to read food and medicine labels, and how to make healthy food choices; health skills that learners showed quantitative increases in confidence performing and engaged with the most. Students from non-English speaking backgrounds reported translating the health information they had learnt for the benefit of those less proficient in English.

*“… Sometimes I tell my friend, I tell my relative ‘… don't buy this one for not good for healthy’, I help someone. Because I live Cabramatta [suburb] someone don't know… I tell them ‘don’t eat too much this one, it’s no good’. Because I speak Cantonese and Vietnamese and Mandarin, I can explain for, helping everyone.” (S120. F, 52, NESB)*

#### Attitudes

Issues of health became more prominent for students who often discussed their increased motivation and desire to be healthy, for longevity and better quality of life.

*“…we have to know … how to keep the body always healthy, this a lot important because I don’t want to get sick. I want to have the long life, that’s very important, how to balance our life with the good food and then with the exercise…” (S101, F, 48, NESB)*

#### Communication with healthcare professionals

Students felt better able and more confident to communicate with healthcare professionals. They were able to communicate information about symptoms and specific health conditions using specific strategies such as diagrams and writing out specific terms prior to consultation. They reported having a greater ability to elicit information from healthcare providers by asking questions.

*“When I go for talk to my Doctor I can explain, I ask him more about my problems. When I go the pharmacy, ask the pharmacist how…to use the medication…I make change a lot because I am feeling more confident when I go the Doctor, when I go the pharmacist, I no quiet…” (S114, M, 56, NESB)*

*“Sometime I have problem, symptom…I want talk with Doctor. Now I feel better because teacher give me the … human body. I write out the word … I bring the picture, show them.” (S120, F, 52, NESB)*

#### Broader benefits of the course

The benefits of the course extended beyond the health domain. Students and teachers both commented on class members’ improved vocabulary and English language skills over the duration of the course.

*“… they built up their vocabulary, a greater understanding. Especially when we talked about different health conditions so becoming familiar with those names and what they actually meant.”(T202)*

Students and teachers also expressed that the course built student confidence to engage in future learning, seek employment and deal with external stressors.

*“Yes because if it wasn’t for this course there was no way I was going to go for a job, no way, especially for this job… I don’t have to be stressed out or you know, I can do all that stuff.” (S107, F, 47, NESB)*

The program allowed students to meet new people and develop social confidence and skills to interact with classmates and communicate in different settings.

*“I was happy to, to go there and also I, meet people from there.... and they were…enjoying the course with me as well” (S106, M, 55, NESB).*

*“Yeah, their confidence changed, they became more confident in talking within a group.” (T202)*

### Student engagement and perceptions of the course

Students enjoyed learning about health and found the range and content of topics engaging, particularly topics concerning food. They valued the patience shown by the teachers and their willingness to reiterate information and clarify concepts which were not initially understood.

*“The teacher I had she really explained to us, you know…The program was good which was organised and clear … I’m really impressed with this course.” (S107, F, 47, NESB)*

Teachers commented that the course seemed to captivate students, and increase their attention for learning and developing knowledge and skills. Students were highly engaged with class activities.

*“Oh, very interesting [referring to the course]… I could tell by the way that they engaged with the topics, their discussions. We spent a lot of time on discussion and doing the activities, they just loved it, it was very interesting… Fully engrossed the whole time, I was amazed.” (T202)*

*“…attendance was excellent, engagement in activities was really, really good…” (T201)*

### Challenges in participation and delivery

Students experienced some challenges, most frequently relating to understanding specific health-related content and concepts and learning to use subject-specific terminology. Individual strategies (e.g. using dictionaries to define new words) and class strategies (e.g. continuous class reinforcement), were developed to overcome the identified challenges.

*“Yes I like but…sometime very difficult understanding … We have a lot of new meanings and new words them a little bit hard, yeah.” (S103, M, age not given, NESB)*

*“Some was a little bit hard for them and…it moved pretty quickly and so we had to do a few things that would just add to the revision…we did things like they created their own find-a-words and I also created for them activities with the Smart-Board games…” (T201)*

### Teaching experience and recommendations

Teachers commented that resources were easy to deliver, with thorough explanations and that the topic of health was not a barrier to delivery. One teacher commented that she was able to grow in confidence in delivering health content as the course progressed.

*“Look, the resource is excellent, seriously is and the explanations are really full. It’s, it was easy to deliver, to get together”. (T201)*

*“I grew in confidence as I went along because I hadn’t delivered any, anything on health in depth before so I had some learning to do as well… I think the program was excellent” (T202)*

Teachers appeared to embrace the flexibility inherent in the course structure and opted to skip particular topics (e.g. budgeting) which they felt were not relevant to their cohort.

*“I didn’t do a lot with the budgeting because I actually knew my students, knew by that stage that they didn’t really need that…” (T201)*

Teachers also offered a range of specific suggestions on how to improve the program for future iterations. See Table 6.

**Table 6:** Specific suggestions for program improvement provided by teachers

- Include Smart Board (an interactive electronic learning resource) activities for revision.
- Include more gender-neutral examples.
- Include internet links to find further information.
- Use images which are more relevant to an Australian context.
- Divide the assessments into smaller sections to be completed over several days.

## Discussion

This study investigated the feasibility of teaching health literacy in an Australian adult education program for socially disadvantaged adults. The *Being Healthy, Staying Healthy* program was successfully delivered within an established course structure at 2 adult education sites in New South Wales, Australia in 2012. The program had high rates of participation and retention. Teachers commented that the program was easy to deliver and the content was deemed appropriate and engaging for basic/beginner (Australian Core Skills Framework stage 2) adult learners. Quantitative and qualitative findings suggest a health literacy program could positively impact functional health skills and knowledge, and communication with healthcare providers. They also shed light on the utility of different measurement instruments in an adult education context.

The *Being Healthy, Staying Healthy* program successfully integrated health content into an established basic/beginner (stage 2) Learning, Literacy and Numeracy course at two sites by providing teaching manuals with flexible lesson plans and resources. This approach received institutional and staff support; the study shows that it was acceptable to adult literacy teachers regardless of the different course structures of the sites. Teachers valued the full explanations provided within the resource and were able to grow in confidence delivering health content as the course progressed. This appears be an appropriate and feasible approach to engage adult education institutions in a larger Australian trial which would involve teachers with varied experience developing or delivering health content and varied course delivery structures.

This study had 100 % participation, supporting the feasibility of our recruitment approach. The same method will be adopted for the larger trial. The study also had a retention rate above 90 % (albeit a small sample) for the 80–90 hours of the program. It is typically very difficult to retain adult learners for extended periods of time, and attrition has been described as the most important problem in adult basic education [30]. In 2000, the Massachusetts Institute for a New Commonwealth analysis of Adult Education, *New Skills for a New Economy*, reported one in five learners leave adult basic education before they have completed 25 hours of instruction and only 21 % receive at least 150 hours [31]. Health is a powerful catalyst for learning [32]. It is part of every learner’s personal experience and engages their interest because the information is immediately applicable for learners or their families [32]. Functional Context Education methods is founded on the principle that making explicit the relationship between what students want to learn, what is being taught, and its application in the contexts that the person will be functioning in, will promote increased motivation [25].

In the study reported here, adult learners expressed satisfaction with the course and interest in, and engagement with, health topics covered. Flexibility in the course structure which allowed teachers and students to choose many of their topics to address specific deficits in health knowledge or areas of interest, may have encouraged learner persistence. In a larger trial with greater geographic scope, the demographic composition of campuses will vary widely. Maintaining a flexible structure will be important to sustain high satisfaction and retention across campuses which have different ages, language skills, interests or health needs.

This study captured gains in specific functional health literacy skills such as using a thermometer, reading medicine, food and nutrition labels, and understanding healthcare professionals. Qualitative reports also suggest that students were better able to communicate with healthcare professionals about symptoms and medication use. Other health literacy trials have also detected change in the functional health skills of adult learners using purpose designed, curriculum-based measures [22, 24]. Developing specific functional and communicative skills can have positive implications for health outcomes of socially disadvantaged adults. An improved ability to communicate with doctors and read medicine labels, for example, has the capacity to improve medication self-management [33]. The functional health literacy skills assessment and qualitative sub-component appear to be appropriate measures and methods to assess important impacts of the program in the larger trial.

There were no apparent changes on the generic functional, communicative and critical health literacy measure [27]. This measure was developed and validated for a diabetes patient population [27]. Some items (e.g. “In reading instructions or leaflets from hospitals/pharmacies, you found that the print was too small to read”) reflect organisational or contextual determinants of health literacy that may not be amenable to change as a result of individual participation in a health literacy program. For other items (e.g. “…You have understood the obtained information”), it may be impracticable to expect to detect a change within one study semester. A recently developed measure, the Health Literacy Questionnaire, attempts to capture the multidimensional nature of health literacy [34]. The scales which comprise it cover a broad range of issues which can be interpreted as intrinsic or extrinsic dimensions of health literacy, and researchers can select scales appropriate to the context and purpose of their study. We will replace the current measure with the Health Literacy Questionnaire in the larger trial.

We will also attempt to develop and measure more advanced health literacy skills in the larger trial. Our qualitative findings suggest that these skills (e.g. communicative health literacy skills) can be developed through such programs. Existing health literacy programs have focused on a limited set of functional health literacy skills, such as reading a thermometer, which may not position students to effectively generalise their learning to other skills and areas. Shared decision making (SDM) is an important aspect of communicative and critical health literacy. We will incorporate a SDM component into the larger health literacy trial to promote skills to obtain relevant health information, derive meaning and apply information, and share decision-making with healthcare professionals [12]. Developing communicative and critical health literacy skills in health literacy courses may be key to expanding the effectiveness and generalizability of health literacy training in a way which can be meaningfully captured by measures such as the Health Literacy Questionnaire.

The majority of participants in the *Being Healthy, Staying Healthy* program were from culturally and linguistically diverse backgrounds and spoke a language other than English. Low literacy and poor English language skills are distinct. Whilst addressing adult low literacy requires teaching someone to read and write, spoken communication and vocabulary are relatively straightforward. On the other hand, addressing poor English language skills requires additional attention to these elements, and students may be highly literate in their native language. The larger trial can expect to recruit both native English speakers and those from non-English speaking backgrounds, with implications for implementation and evaluation. Challenges pertaining to understanding and learning terminology and vocabulary, for example, should be less significant for native English speakers who have stronger English language skills. The program is flexible enough to enable teachers to adapt resources for their cohort, such as incorporating English definition exercises or adding more complex writing tasks. Optional language revision activities using interactive electronic learning resources developed from the pilot (Table 6) will be shared in the larger trial and qualitative methodologies with purposive sampling will be employed to understand the experiences of diverse campuses.

## Conclusion

Enhancing health literacy in socially-disadvantaged adults is an important endeavor needed to address health inequalities. Building community capacity through tailored health literacy courses offers promise in an Australian context where few initiatives exist. Findings from the *Being Healthy, Staying Healthy* study suggest that health literacy courses delivered through adult education settings could be a feasible and engaging mechanism to increase the health skills and knowledge of Australian adults with lower literacy. Given the national scope of adult education efforts, comprehensively understanding the potential impact such courses could have represents an important step in an ongoing effort to address health literacy and health inequalities in Australia.

## Abbreviations

ES, English Speaking; ESL, English as a Second Language; M, mean; NESB, Non-English-Speaking background; NSW, New South Wales; SD, Standard deviation; SDM, Shared Decision Making; S-TOFHLA, Short Test of Functional Health Literacy in Adults; TAFE, Technical and Further Education.

## Additional file

Additional file 1: Functional health literacy skills assessment and marking scheme. (DOCX 453 kb)
