# Supplementary material for: Incorporating health literacy in education for socially disadvantaged adults: an Australian feasibility study
Source: Int J Equity Health. 2016 Jun 4;15:84. doi: 10.1186/s12939-016-0373-1 (PMC4893249; doi:10.1186/s12939-016-0373-1)
Supplement: Additional file 1: — Functional health literacy skills assessment and marking scheme. (DOCX 453 kb) [file 12939_2016_373_MOESM1_ESM.docx]

**Additional file 1: Assessment measures and marking scheme**

**Core knowledge domain: Taking temperatures**

| Thermometer 1 (HS1) | 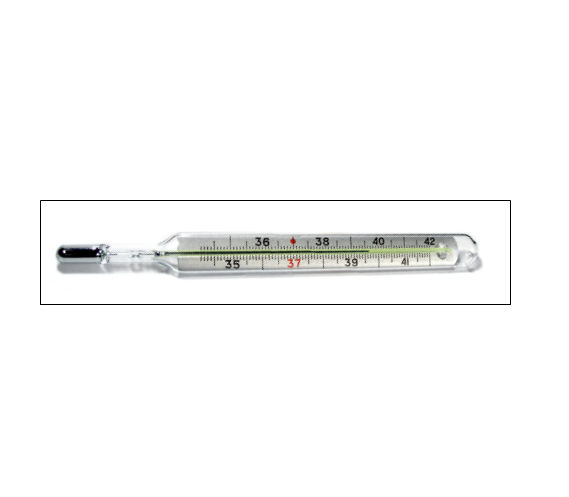 |
| --- | --- |
| Thermometer 2 (HS2) | 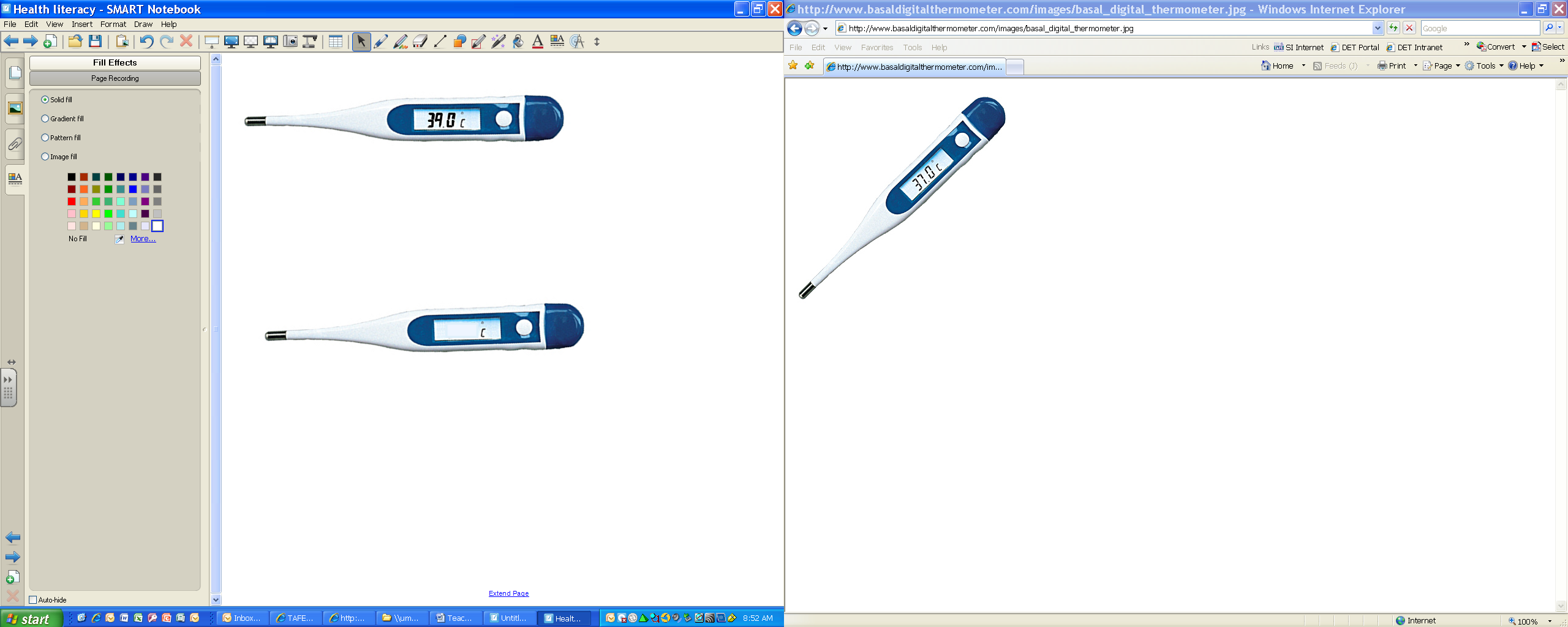 |
| Thermometer 3 (HS3) | 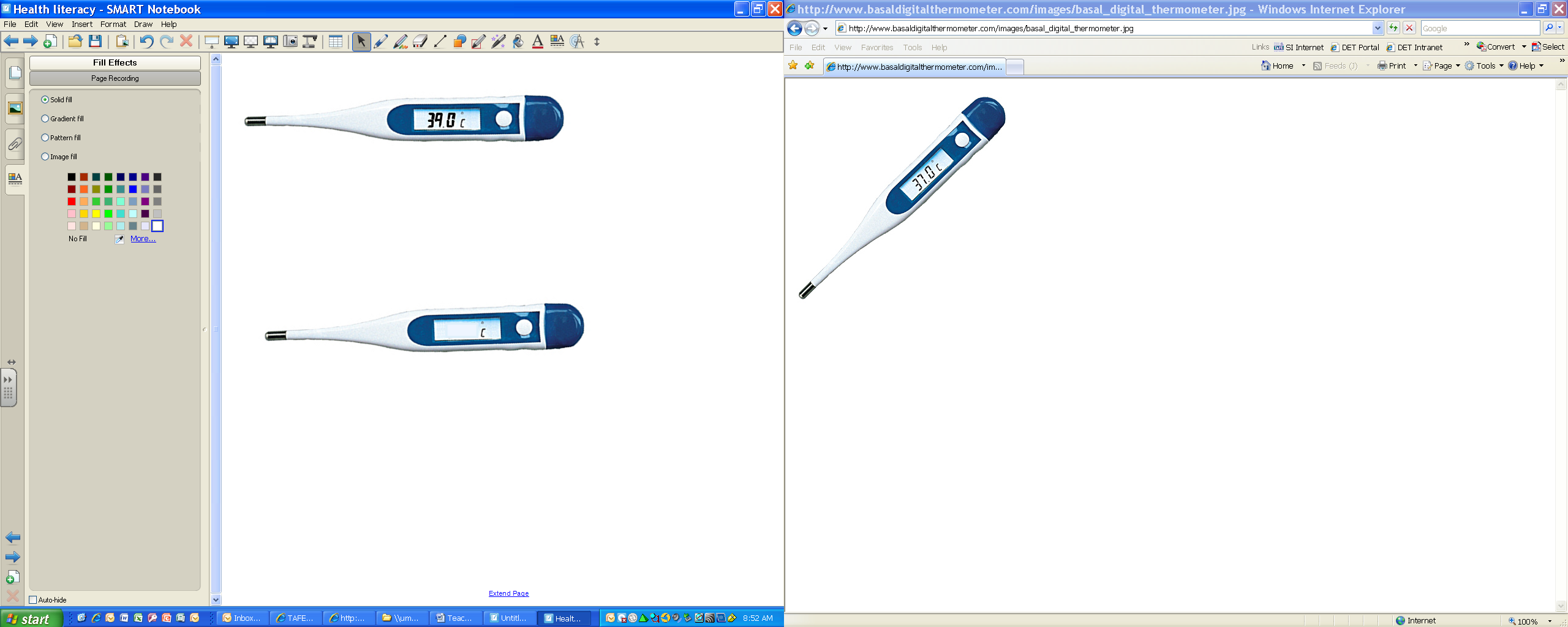 |
| *Figure 1.* Images used for Taking Temperatures Q1-3. | |

| **USE DULLPAIN FOR** | | | 24 MINI CAPSULES | **Dullpain** | 24 | MINI CAPSULES | **Dullpain 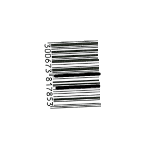** | 24 MINI CAPSULES | **Dullpain** |
| --- | --- | --- | --- | --- | --- | --- | --- | --- | --- |
| ✓headaches and fevers  ✓body aches and pains  ✓toothaches  **Suitable for:**  ✓People with stomach ulcers  ✓People sensitive to aspirin  ✓Breastfeeding mothers | | |  |  |  |  |  |  |  |
| **HOW TO USE DULLPAIN** | | |  |  | Effective pain relief  Gentle for stomachs  Each capsule contains PARACETAMOL 500 mg |  |  |  |  |
| **AGE** | **CAPSULES** | **HOW OFTEN** |  |  |  |  |  |  |  |
| 12- ADULT | 1-2 | Every 4-6 hours  MAXIMUM  8 capsules in 24 hrs |  |  |  |  |  |  |  |
| 7-12 | 1 | Every 4-6 hours  MAXIMUM  4 capsules in 24 hrs |  |  |  |  |  |  |  |
| **DO NOT USE DULLPAIN** | | | 122398 EXP MAY 16 |  |  | Easier to swallow  Gentle for stomaches |  | **ONLY USE IF ALL SEALS ARE INTACT** | ✓Safety sealed  ✓Heat sealed blister pack  ✓Safety seal on carton |
| *X* For children under 7 years  *X* If any seals on packaging are broken  *X* If using other medicines containing paracetamol  *X* For more than 24 hrs for children 7-17 except on medical advice  *X* For more than a few days in adults except on medical advice | | |  |  |  |  |  |  |  |
| **EACH CAPSULE CONTAINS** | | |  |  |  |  |  |  |  |
| - Paracetamol 500 mg - No gluten, lactose or sugar | | |  |  | 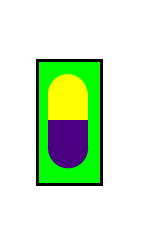 |  |  |  | 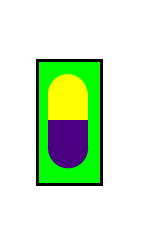 |
| **CAUTION** | | |  |  |  |  |  |  |  |
| If symptoms persist, see your doctor.  If an overdose is taken or suspected, ring the Poisons Information Centre (Aust:131 126) or go to hospital immediately. Overdose can lead to serious liver damage if left untreated. | | |  |  |  |  |  |  |  |
| *Figure 2.* Image used for Medicine Labels Q1-5. | | | | | | | | | |

**Core knowledge domain: Medicine labels**

**Core knowledge domain: Food labels and nutritional information**

|  | | | | | | |
| --- | --- | --- | --- | --- | --- | --- |
| **Purse size sachets** | ***Great for work. Great for play.***  ***Use any time of day.*** | | | **50g** | | |
|  |  |  |  | **2 Sachets** | | |
|  | | | | | | |
| **Ingredients** |  | | **NUTRITION INFORMATION** | | | |
| Vegetables, creamer [vegetable oil, glucose syrup, milk protein, mineral salts (339, 450)], chives, maltodextrin (from wheat), salt, flavour enhances (621, 627), wheat fibre, mineral salt (potassium chloride), vegetable oil, garlic, hydrolysed corn protein, flavours, emulsifier (471), colour, spice extract, preservative (222), food acid (citric) | | | Servings per package: 2  Serving size: 300mL | | | |
|  |  |  |  | | Avg Qty per Serving | Avg Qty per 100mL |
| **Directions**   1. **Empty contents of one sachet into a 300 mL mug.** 2. **Fill with boiling water and stir for 15-20 seconds.** | | **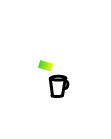**  **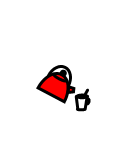**  **Best Before 12/04/13** | Energy  Protein  **Fat, Total**  -saturated  Carbohydrate  -sugar  Dietary fibre  Sodium  Potassium | | 493kJ  2.5g  **2.4**  2.2g  15.6g  1.2g  1.7g  595mg  450mg | 164kJ  0.8g  **0.8g**  0.7g  5.2g  0.4g  0.6g  198mg  150mg |

*Figure 3.* Image used for Food Labels and Nutritional Information Q2-10.

**Core knowledge domain: Oral communication**

Pharmacist: Mrs Smith, could you just confirm your address please? Thank you. Now, there’re two lots of tablets here. These ones are to be taken with water after a meal, three times a day. Take these smaller ones twice a day, preferably on an empty stomach or several hours after eating. Oh, by the way, you might find one of those leaflets over there in the rack useful. OK, bye no
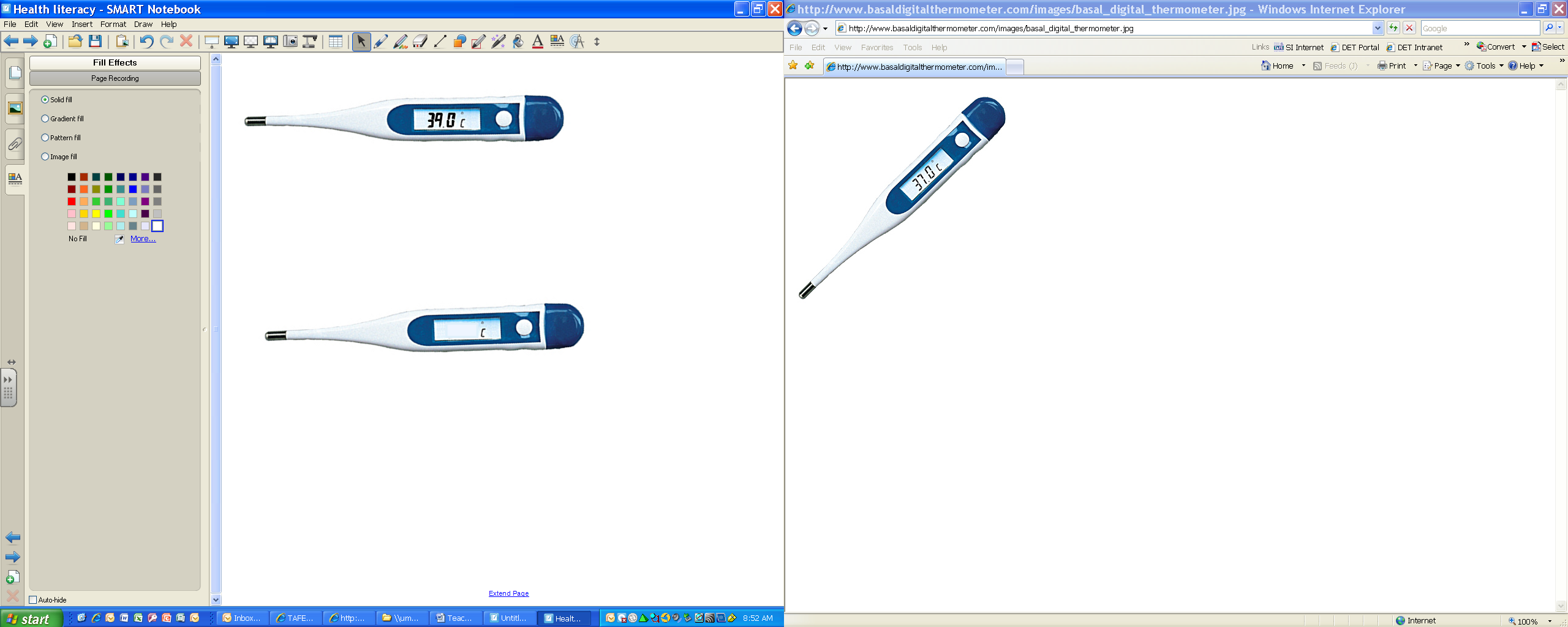
w

| Table 1.  *Marking scheme for BHSH knowledge measure* | | | | | |
| --- | --- | --- | --- | --- | --- |
| **Core knowledge domain** | **Questions [variable code]** | **Correct answer(s)** | **Marking/scoring scheme** | **Maximum mark awarded** |  |
| 1. Taking temperatures | 1. [HS1] What temperature is shown on thermometer 1? | 39.6 | 39 – 40 = 1 mark  Zero for any other values | 1 |  |
|  | 1. [HS2] This morning I took my temperature with thermometer 2. This is the reading I got. It this okay? | No | No = 1 mark  Zero for any other responses | 1 |  |
|  | 1. [HS3] On thermometer 3 write what temperature you think is normal for a healthy adult. | 35.8 – 37.4, generally accepted as 37 | 35.8 – 37.4 = 1 mark  Zero for any other values | 1 |  |
| **SUBTOTAL 1** |  |  |  | **3** |  |
| 1. Medicine labels | 1. [ML1] When does the medicine label expire? | May 2013 | May 2013 = 1 mark  Zero for any other values | 1 |  |
|  | 1. [ML2] I have a stomach ulcer. Can I use this medicine? | Yes | Yes = 1 mark  Zero for any other responses | 1 |  |
|  | 1. [ML3] My son is 6 years old. Can he use this medicine? | No | No = 1 mark  Zero for any other responses | 1 |  |
|  | 1. [ML4] I took 2 capsules at 10am. When can I take another 2? | 2pm – 4pm | Any time inclusive of 2pm – 4pm = 1 mark  Zero for any other responses | 1 |  |
|  | 1. [ML5] My 3 year old son found a packet and has eaten 6 capsules. What should I do? | Call the Poison Information Centre or go to hospital immediately | Similar to correct = 1 mark | 1 |  |
| **SUBTOTAL 2** |  |  |  | **5** |  |
| 1. Food labels and nutritional information | 1. Do you look at food labels when you buy packaged food? | Yes/No | NA | NA |  |
|  | 1. [FNL1] Can you tell me what this packaged food is? | Use of words such as soup, potato and leek soup, vegetable soup, dried, sachet | Similar to correct = 1 mark  Zero for any other responses | 1 |  |
|  | 1. [FNL2] Can you tell me what the main ingredient of this soup is? | Vegetables (first in list) | Same as correct = 1 mark | 1 |  |
|  | 1. [FNL3] Can you tell me how to make this soup? | Empty contents of one sachet into a 300 mL mug. Fill with boiling water and stir for 15-20 seconds | Similar to correct answers.  Empty contents of one sachet into a (300 mL) mug= 1 mark.  Fill with boiling water and stir for 15-20 seconds= 1 mark. | 2 |  |
|  | 1. [FNL4] If this package was being sold next July would you buy it? | No. Because past the best buy date or never buy packaged food or food with preservatives /colouring /flavouring. | Similar to correct answers (2 marks)  No= 1 mark  Reason why = 1 mark | 2 |  |
|  | 1. [FNL5] Do you know what kilojoules are? | A measurement of energy. Or explain that food high in kilojoules are high energy foods or can lead to weight gain. Or give examples of high kilojoule foods such as butter, fats, carbohydrates and sugar. | Similar to correct answers. | 1 |  |
|  | 1. [FNL6] How many kilojoules are in a serve of this soup? | 493 kj | 493 kj = 1 mark  Zero for any other value | 1 |  |
|  | 1. [FNL7] How do you find out if this food is high in salt? | Look at sodium in the nutrition information table | Look at sodium or nutrition information table = 1 mark  Zero for any other response | 1 |  |
|  | 1. [FNL8] Why would this be important to check? | Salt/sodium can cause high blood pressure | Similar to correct answer = 1mark | 1 |  |
|  | 1. [FNL9] What else would you check to make sure this food was healthy? | Any of the following: fat, sugar, any allergy foods (nuts, dairy, wheat, gluten), added flavours/ colours/preservatives/ numbers in the ingredients. | Correct if response is similar to correct answers listed = 1mark | 1 |  |
| **SUBTOTAL 3** |  |  |  | **11** |  |
| 1. Oral communication | 1. [OC1] There are ___ lots of tablets | 2 | Correct answer of 2 = 1 mark | 1 |  |
|  | 1. [OC2] For the first tablets, you take ___ times a day, ___ or ___ meals | 3, with water after OR before or after | Correct answers = 2 marks | 2 |  |
|  | 1. [OC3] The smaller tablets are taken ___ times a day on an ___ | 2, empty stomach/ several hours after eating | Correct answers = 2 marks | 2 |  |
|  | 1. [OC4] What could you do if you did not understand the pharmacist? | Any of the following: ask him to repeat, repeat what he said so he could agree or disagree or ask questions to check how much and when to take medicine | Similar to correct answer= 1 mark | 1 |  |
| **SUBTOTAL 4** |  |  |  | **6** |  |
|  |  |  | **Maximum Total Score** | **25** |  |
